# MRI evaluation of multifocality and contralateral disease in lobular breast cancer: what can we learn from one region's experience?

**DOI:** 10.1186/bcr3770

**Published:** 2015-11-05

**Authors:** Claire Magee, Keith Lowry

**Affiliations:** 1 Belfast City Hospital, Belfast Health and Social Care Trust, Belfast, UK; 2 NIMDTA, Belfast, UK

## Introduction

Invasive lobular cancer has been associated with an increased risk of multifocal and contralateral disease. The literature suggests an incidence of contralateral disease as high as 15%. Current national (NICE) recommendations are that all patients with lobular carcinoma being considered for breast-conserving surgery have a preoperative breast MRI. The objective was to identify the rate of additional MRI-detected multifocal and contralateral disease in patients with a newly diagnosed lobular cancer to determine whether it is as high as the literature suggests. Based on our findings we hope to further explore whether another imaging alternative should be considered.

## Methods

A retrospective search was done on PACS to identify all those patients in Northern Ireland investigated with bilateral breast MRI for a newly diagnosed cancer during a 15-month period. MRI findings were correlated with histopathology records from all regional labs and the data analysed.

## Results

A total of 141 patients had an MRI for biopsy-proven lobular carcinoma. Within this regional cohort the incidence of additional contralateral and multifocal disease was 2.13% and 13.4% respectively.

## Conclusions

The incidence of contralateral lobular disease is 2.13%, within our reasonably large study population, significantly less than the currently cited 15%. Our study does show a significant increase in detection of multifocal disease in the same breast by MRI. Based on our results consideration should be given to exploring the use of tomography or contrast-enhanced mammography prior to MRI to attempt to detect further disease. This could potentially expedite patient care.

